# Combinatorial immune checkpoint blockade increases myocardial expression of NLRP-3 and secretion of H-FABP, NT-Pro-BNP, interleukin-1β and interleukin-6: biochemical implications in cardio-immuno-oncology

**DOI:** 10.3389/fcvm.2024.1232269

**Published:** 2024-01-23

**Authors:** V. Quagliariello, M. Passariello, I. Bisceglia, A. Paccone, A. Inno, C. Maurea, R. Rapuano Lembo, L. Manna, M. Iovine, M. L. Canale, M. Scherillo, P. A. Ascierto, D. Gabrielli, C. De Lorenzo, N. Maurea

**Affiliations:** ^1^ Division of Cardiology, Istituto Nazionale Tumori-IRCCS-Fondazione G. Pascale, Naples, Italy; ^2^ Department of Molecular Medicine and Medical Biotechnology, University of Naples “Federico II”, Naples, Italy; ^3^ Servizi Cardiologici Integrati, Dipartimento Cardio-Toraco-Vascolare, Azienda Ospedaliera San Camillo Forlanini, Rome, Italy; ^4^ Medical Oncology, Istituto di Ricovero e Cura a Carattere Scientifico (IRCCS) Ospedale Sacro Cuore Don Calabria, Negrar, Italy; ^5^ Medical Oncology, Ospedale del Mare, Naples, Italy; ^6^ Department of Molecular Medicine, Ceinge-Biotecnologie Avanzate s.c.a.r.l., Naples, Italy; ^7^ U.O.C. Cardiologia, Ospedale Versilia, Lido di Camaiore (LU), Camaiore, Italy; ^8^ Cardiologia Interventistica e UTIC, A.O. San Pio, Presidio Ospedaliero Gaetano Rummo, Benevento, Italy; ^9^ Melanoma Cancer Immunotherapy and Innovative Therapy Unit, Istituto Nazionale Tumori IRCCS Fondazione "G. Pascale", Naples, Italy; ^10^ U.O.C. Cardiologia, Dipartimento Cardio-Toraco-Vascolare, Azienda Ospedaliera San Camillo Forlanini, Roma – Fondazione per il Tuo Cuore – Heart Care Foundation, Firenze, Italy

**Keywords:** cancer, cardiotoxicity, immune checkpoint inhibitors, myocarditis, inflammation, oncology, cardiology

## Abstract

**Background:**

Immune checkpoint blockade in monotherapy or combinatorial regimens with chemotherapy or radiotherapy have become an integral part of oncology in recent years. Monoclonal antibodies against CTLA-4 or PD-1 or PDL-1 are the most studied ICIs in randomized clinical trials, however, more recently, an anti-LAG3 (Lymphocyte activation gene-3) antibody, Relatlimab, has been approved by FDA in combination with Nivolumab for metastatic melanoma therapy. Moreover, Atezolizumab is actually under study in association with Ipilimumab for therapy of metastatic lung cancer. Myocarditis, vasculitis and endothelitis are rarely observed in these patients on monotherapy, however new combination therapies could expose patients to more adverse cardiovascular events.

**Methods:**

Human cardiomyocytes co-cultured with human peripheral blood lymphocytes (hPBMCs) were exposed to monotherapy and combinatorial ICIs (PD-L1 and CTLA-4 or PD-1 and LAG-3 blocking agents, at 100 nM) for 48 h. After treatments, cardiac cell lysis and secretion of biomarkers of cardiotoxicity (H-FABP, troponin-T, BNP, NT-Pro-BNP), NLRP3-inflammasome and Interleukin 1 and 6 were determined through colorimetric and enzymatic assays. Mitochondrial functions were studied in cardiomyocyte cell lysates through quantification of intracellular Ca^++^, ATP content and NADH:ubiquinone oxidoreductase core subunit S1 (Ndufs1) levels. Histone deacetylases type 4 (HDAC-4) protein levels were also determined in cardiomyocyte cell lysates to study potential epigenetic changes induced by immunotherapy regimens.

**Results:**

Both combinations of immune checkpoint inhibitors exert more potent cardiotoxic side effects compared to monotherapies against human cardiac cells co-cultured with human lymphocytes. LDH release from cardiac cells was 43% higher in PD-L1/CTLA-4 blocking agents, and 35.7% higher in PD-1/LAG-3 blocking agents compared to monotherapies. HDAC4 and intracellular Ca^++^ levels were increased, instead ATP content and Ndufs1 were reduced in myocardial cell lysates (*p* < 0.001 vs. untreated cells). Troponin-T, BNP, NT-Pro-BNP and H-FABP, were also strongly increased in combination therapy compared to monotherapy regimen. NLRP3 expression, IL-6 and IL-1β levels were also increased by PDL-1/CTLA-4 and PD-1/LAG-3 combined blocking agents compared to untreated cells and monotherapies.

**Conclusions:**

Data of the present study, although *in vitro*, indicate that combinatorial immune checkpoint blockade, induce a pro- inflammatory phenotype, thus indicating that these therapies should be closely monitored by the multidisciplinary team consisting of oncologists, cardiologists and immunologists.

## 1 Introduction

The recent combined immune checkpoint (ICI) inhibition therapy with Ipilimumab (a CTLA-4 blocking agent) and Nivolumab (a PD-1 blocking agent) demonstrated better anticancer outcomes in advanced or metastatic melanoma compared to monotherapy regimen (1). More recently, an anti-PD-L1 blocking agent, called Atezolizumab, in combination with Ipilimumab is currently under evaluation for metastatic non small cell lung cancer therapy (2). The concept of combined immune checkpoint blockade is growing for therapy of various tumors with intra-tumor lymphocytes (3). A simultaneous inhibition of two pathways having a key role in the induction of peripheral immune tolerance optimizes recognition and antitumor efficacy of specialized lymphocytes (4). Lymphocyte activation gene-3 (LAG-3) is another immune checkpoint receptor which can negatively regulate T cell functions by competing with CD4 for binding to MHC-II (5). In brief LAG-3 is able to enhance regulatory T-lymphocytes levels through IL-10, resulting in a significant increase of cancer cells immune escape (6); therefore LAG-3 it is considered a new target of great clinical interest in cancer immunotherapy (7). The first anti-LAG3 human monoclonal antibody, Relatlimab (Bristol-Myers Squibb), has been recently approved (March 2022) by FDA in combination with Nivolumab for metastatic melanoma therapy (8) and is currently under study in 46 randomized clinical trials in oncology. Notably, LAG-3 blocking agents are currently studied in combinatorial therapy regimen with CTLA-4 or PD-1 monocloncal antibodies.

Immune-related adverse events, including endocrinopathies, mucositis, gastritis, arthritic pain and dermatitis could induce discontinuation therapy in just under 50% of patients (9, 10); however, these events are currently clinically managed through antihistamines, anti-inflammatory drugs, glucocorticoids, including prednisolone and methylprednisolone and others (11). Although rare, ICIs-related cardiovascular events can occur in cancer patients (12). Events of inflammatory myocarditis, vasculitis and endothelitis are seen in clinical practice and some of them may be at high mortality (12, 13). Non-viral inflammatory myocarditis is the most observed cardiotoxic event in patients on ICIs, however the frequency is extremely low (<1%) (14). Patients treated with pembrolizumab or ipilimumab may also develop fulminant myocarditis with fatal outcomes, however in CTLA-4/PD-1 blocking agents association studies show an increase in myocarditis events compared to monotherapies (15). Furthermore, it is not easy to diagnose myocarditis and many studies probably underreport these cardiotoxic events (16). The diagnostic screening of myocarditis and vasculitis is not always easy and possible, therefore an underestimation of the real cases of ICIs-related cardiac events is very likely (17). Based on this, great international attention has been given to the early and effective diagnosis of ICIs-induced myocarditis and vasculitis and to the profound study of the mechanisms of cardiovascular immunotoxicity. Recent international guidelines from the AHA and ESC provided data on the mechanisms of immune toxicity that includes pro-inflammatory cytokines and granzyme-B produced by hyperactivated lymphocytes in myocardial tissue (18). Recent works have established that NLRP3 and MyD-88 may be key drivers of ICIs-induced myocarditis and that some metabolic factors such as hyperglycemia and visceral obesity may worsen the outcome (19, 20).

Recent American Heart Association statements suggest a surveillance strategy for ICIs-related cardiovascular side effects at baseline with electrocardiogram, BNP or NT-Pro-BNP, cardiac troponin and echocardiogram (18, 21). At high risk patients, AHA suggests quantification of BNP or NT-Pro-BNP and troponin, before ICIs and at 2, 3 and 4 doses of therapy. These suggestions lead to great attention towards patients treated with ICI. In light of the new combinatorial immune checkpoint blockade therapies, basic pathophysiological studies are needed to predict possible adverse events in clinic scenario. This study focalized, for the first time, on the potential cardiotoxic side effects of novel combinatorial ICIs treatments on models of lymphocyte-cardiomyocyte interaction, such as cardiomyocyte/hPBMCs co-cultures (22), through selective analysis of cardiac cell lysis and quantification of several pro-inflammatory and conventional cardiotoxic biomarkers.

## 2 Materials and methods

### 2.1 Co-culture of hPBMC and HFC

**Figure 1:**
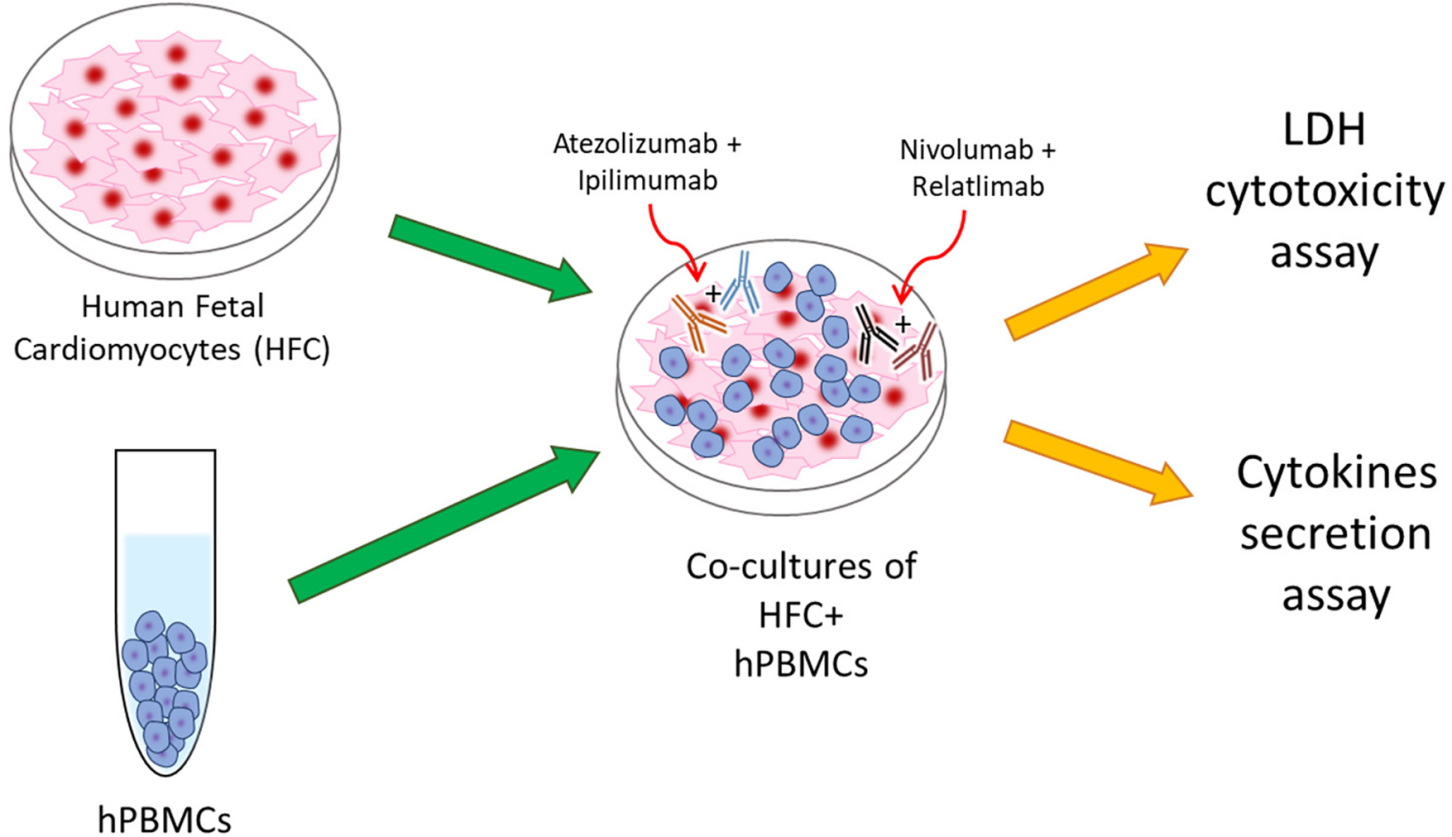
Schematical illustration of HFC + hPBMCs co-cultures as model of ICIs-mediated cardiotoxicity due to direct interaction between cardiac and immune cells.

Briefly, human cardiac cells, called HFCs (Innoprot, Derio, Spain), were cultured in a selective culture medium + Fetal Bovine Serum (FBS 10%v/V, Sigma Aldrich, St. Louis MO, USA), Penicillin at 50 U/ml, Streptomycin at 50 μg/ml and L-Glutamine at 1%v/V. Cardiomyocytes were than plated in a 96 well plates (10,000 cells/well) for 16 h. Human Peripheral Blood Mononuclear Cells (hPBMCs) were added at effector: target ratio of 5:1 with and without ICIs in monotherapy or combined regimen, such as Relatlimab or Nivolumab (or both combined) and Atezolizumab or Ipilimumab (or both combined), all used at 100 nM and incubated for 48 h at 37 °C, as described in other experimental research (19, 20) (Figure 1). ICIs molar concentration reflects the dose reached in patient serum/plasma administered with the conventional therapeutic doses of immunomodulatory mAbs (10 mg/kg) (23). Control groups were identified as cells untreated or treated with a standard IgG. Notably, experimental conditions including ratio of cellular components are in line with recent established work of our and other group that mimic lymphocyte-cardiomyocyte interaction in preclinical and clinical models (19, 20, 23).

### 2.2 LDH assay

As demonstrated in our previous work (23), considering the expression of PD-L1, PD-1 and CTLA-4 on cardiomyocytes, after treatment with antibodies described in paragraph 2.1, lymphocytes were removed (collecting the supernatant of co cultures). Cardiac cell lysis was determined through the quantification of released LDH (LDH detection kit, Thermo-Fisher Scientific, Meridian Rd., Rockoford, IL, USA), following the manufacturer's instructions. The sensitivity of this method was below 0.01 OD for absorbance at 490 and 680 nm, and the assay accurately detected LDH release in the range of 0.01–0.4 OD. Reported values refer to a sample of cells exposed exclusively to Triton × 100 lysis solution at 10%v/V.

### 2.3 HDAC4 expression

Histone deacetylases (HDACs) type 4 are enzymes that affect gene expression through acetylation of core histones of chromatin and are involved in pathogenesis of chemotherapy-induced heart failure (24, 25). After treatments described in paragraph 2.1, cardiomyocytes were harvested and lysed through a lysis buffer (1 mM EDTA, 20 mM NaF, 3 mM Na_3_VO_4_, 100 mM NaCl, 1 mM PMSF, 50 mM Tris/HCl, selective protease inhibitor). Cell lysates were centrifuged and supernatants were analyzed through Human HDAC4 ELISA Kit (AB300311, AbCam, Milan Italy) in line with manufacturer's instructions. Control group refers only to untreated cells (Untreated HFC); sensitivity of the ELISA was 94.776 pg/ml and the assay accurately detected HDAC4 expression in the range of 312.5–20,000 pg/ml).

### 2.4 Intracellular Ca^++^, ATP and NADH:ubiquinone oxidoreductase core subunit S quantification assay

Intracellular Ca^++^, ATP content and NADH:ubiquinone oxidoreductase core subunit S expression reflect the mitochondrial activity of cardiomyocytes and their metabolic integrity (26–28). For intracellular calcium quantification, a fluorescence dye Fluo-3 AM, following the manufacturer's protocol, was used. In brief, after treatments described in paragraph 2.1, cardiomyocytes were loaded with 5 μM Fluo-3 AM at 37 °C for 30 min in the dark, and then washed three times with PBS to remove excess of dye. The fluorescence intensity of Fluo-3 chelated with calcium was recorded on a microplate spectrofluorometer (xMark Microplate, Spectrofluorometer Biorad, Milan, Italy) at excitation and emission wavelengths of 488 and 525 nm, respectively. For ATP and Ndufs1 analysis, after treatments described in paragraph 2.1, cardiomyocytes were harvested and lysed through a lysis buffer (1 mM EDTA, 20 mM NaF, 3 mM Na_3_VO_4_, 100 mM NaCl, 1 mM PMSF, 50 mM Tris/HCl, selective protease inhibitor). Cell lysates were centrifuged and supernatants were analyzed through ATP Assay Kit (Colorimetric/Fluorometric) (AB83355, AbCam, Milan, Italy) and Human NADH Dehydrogenase Ubiquinone Fe-S Protein 1 (NDUFS1) ELISA Kit (Abbexa, Uk) in line with manufacturer's instructions. Control group refers only to untreated cells (Untreated HFC); sensitivity of the ATP assay was <1 µM. Sensitivity of the ELISA was <0.12 ng/ml and the assay accurately detected Ndufs1 expression in the range of 0.312 ng/ml–20 ng/ml).

### 2.5 Lipid peroxidation and intracellular oxidative stress

Intracellular reactive oxygen species and lipid peroxidation are key mediators of anticancer drug-induced cardiovascular diseases, including myocarditis (29). In brief, after treatments described in paragraph 2.1, cardiomyocytes were washed three times with cold PBS, harvested with 0.25%v/v Trypsin and centrifuged at 1,000×*g* for 10 min. The supernatant was discarded and the cell pellet sonicated in cold PBS. After a centrifugation step at 800×*g* for 5 min, we quantified intracellular ROS and two markers of lipid peroxidation called malondialdehyde (MDA) and 4-Hydroxynonenal (4-HNA) through commercial kit with a spectrophotometer according to the manufacturer's protocols (Sigma Aldrich, Milan, Italy). According to the kit instructions, the total protein content of cardiac cell homogenates was quantified through Micro BCA protein assay kit (Pierce, Thermo Fisher, Milan, Italy).

### 2.6 NLRP-3 assay

NLRP-3 (inflammasome) is the key driver of cardiovascular diseases and anticancer-drug induced cardiotoxicity (30); therefore, after treatments described in paragraph 2.1, cells were harvested and lysed through a lysis buffer (1 mM EDTA, 20 mM NaF, 3 mM Na_3_VO_4_, 100 mM NaCl, 1 mM PMSF, 50 mM Tris/HCl, selective protease inhibitor). Cell lysates were centrifuged and supernatants were analyzed through NLRP-3 ELISA Kit (code OKEH03368; Aviva Systems Biology, San Diego, CA, USA) in line with manufacturer's instructions (31). Control group refers only to untreated cells (Untreated HFC); sensitivity of the ELISA was 0.078 ng/ml and the assay accurately detected NLRP-3 expression in the range of 0.1–40 ng/ml).

### 2.7 Cytokine panel: quantification of Il-1β, Il-6, TNF-α, Il-4, Il-23, Il-17a, Il-12, Il-18 and INF-γ

Pro-inflammatory cytokines are key players of heart failure and myocarditis (32, 33). As recently described in literature (33) were quantified in supernatant of cardiac cells exposed to combined ICIs therapies through ELISA methods. Briefly, after treatment with Relatlimab or Nivolumab (or both combined) and Atezolizumab or Ipilimumab (or both combined), culture supernatants were centrifuged and analyzed for IL-1β, IL-6, TNF-α, IL-4, IL-23, IL-17a, IL-12, IL-18 and INF-γ quantification through ELISA method in line with literature (31) (Sigma Aldrich, Milan, Italy) in line with manufacturer's instructions (31). Control group refers only to untreated cells (Untreated HFC). In order to confirm whether the release of cytokines is from cardiomyocytes or lymphocytes, cytokine release tests were also performed in *in vitro* culture models of HFCs only and lymphocytes only (results are available in Supplementary Figure 1). Sensitivity of IL-1β Elisa kit: 0.3 pg/ml and refers to a standard curve range: 0.48–500 pg/ml of IL-1β. Sensitivity of IL-6 Elisa kit: 3 pg/ml and refers to a standard curve range: 1.37–1,000 pg/ml. Sensitivity of TNF-α Elisa kit: 30 pg/ml and refers to a standard curve range: 1,56–1,000 pg/ml. Sensitivity of INF-γ Elisa kit: 15 pg/ml and refers to a standard curve range: 20.6–15,000 pg/ml. Sensitivity of IL-4 Elisa kit: 5 pg/ml and refers to a standard curve range: 3.3–200 pg/ml. Sensitivity of IL-23 Elisa kit: 15 pg/ml and refers to a standard curve range: 28.6–7,000 pg/ml. Sensitivity of IL-17 Elisa kit: 0.5 pg/ml and refers to a standard curve range: 1.6–150 pg/ml. Sensitivity of IL-12 Elisa kit: 1 pg/ml and refers to a standard curve range: 0.8–600 pg/ml. Sensitivity of IL-18 Elisa kit: 20 pg/ml and refers to a standard curve range: 24.7–18,000 pg/ml.

### 2.8 H-FABP, NT-Pro-BNP, troponin-T and BNP assay

The heart-type fatty acid binding protein (called H-FABP) is a new putative biomarker of cardiotoxicity (29, 34, 35); in fact, after 30 min of myocardial injury, plasma levels of hFABP are significantly increased (36). After treatments described in paragraph 2.1, cells were harvested and lysed as described in paragraph 2.3. Intracellular levels of H-FABP (AbCam, Cambridge, UK cat: ab243682) were measured through ELISA method in line with literature (37) and manufacturer's instructions. Control group refers only to untreated cells (Untreated HFC). Sensitivity of H-FABP Elisa kit: 2.4 pg/ml and refers to a standard curve range: 10.9–700 pg/ml. The NT-pro-brain natriuretic peptide (NT-proBNP), troponin T and BNP are conventional biomarkers of heart failure and recently included in cardiac monitoring of ICIs-mediated cardiovascular diseases (38, 39). To test and confirm if combinatorial ICIs therapies could induce cardiac damages in lymphocyte-cardiomyocyte models, after treatment with Relatlimab or Nivolumab (or both combined) and Atezolizumab or Ipilimumab (or both combined) cardiomyocytes were tested for NT-proBNP production through Human NT-proBNP ELISA Kit (AbCam, Cambridge, UK cat: ab263877), Troponin T (TNNT1) Human ELISA Kit (EHTNNT1,Thermo Fisher, Milan, Italy), and human BNP ELISA Kit (EHNPPB, Thermo Fisher, Milan, Italy) in line with manufacturer's instructions. Control group refers only to untreated cells (Untreated HFC). Sensitivity of NT-Pro-BNP Elisa kit, Troponin T and BNP: 11.5 pg/ml, 0.35 ng/ml and 14 pg/ml, respectively, and refers to a standard curve range: 21.9–1,400 pg/ml.

### 2.9 Statistical analysis

All analysis were performed in triplicates (*n* = 3). Results were presented as mean ± standard deviation (SD). A paired-*t* test was used to compare different treatments, by using a Sigmaplot software (Systat Software Inc., San Jose, CA, USA). *p* < 0.05 was considered as statistically significant difference between groups.

## 3 Results

### 3.1 *In vitro* cytotoxic side effects of combined immune checkpoint blockade

**Figure 2:**
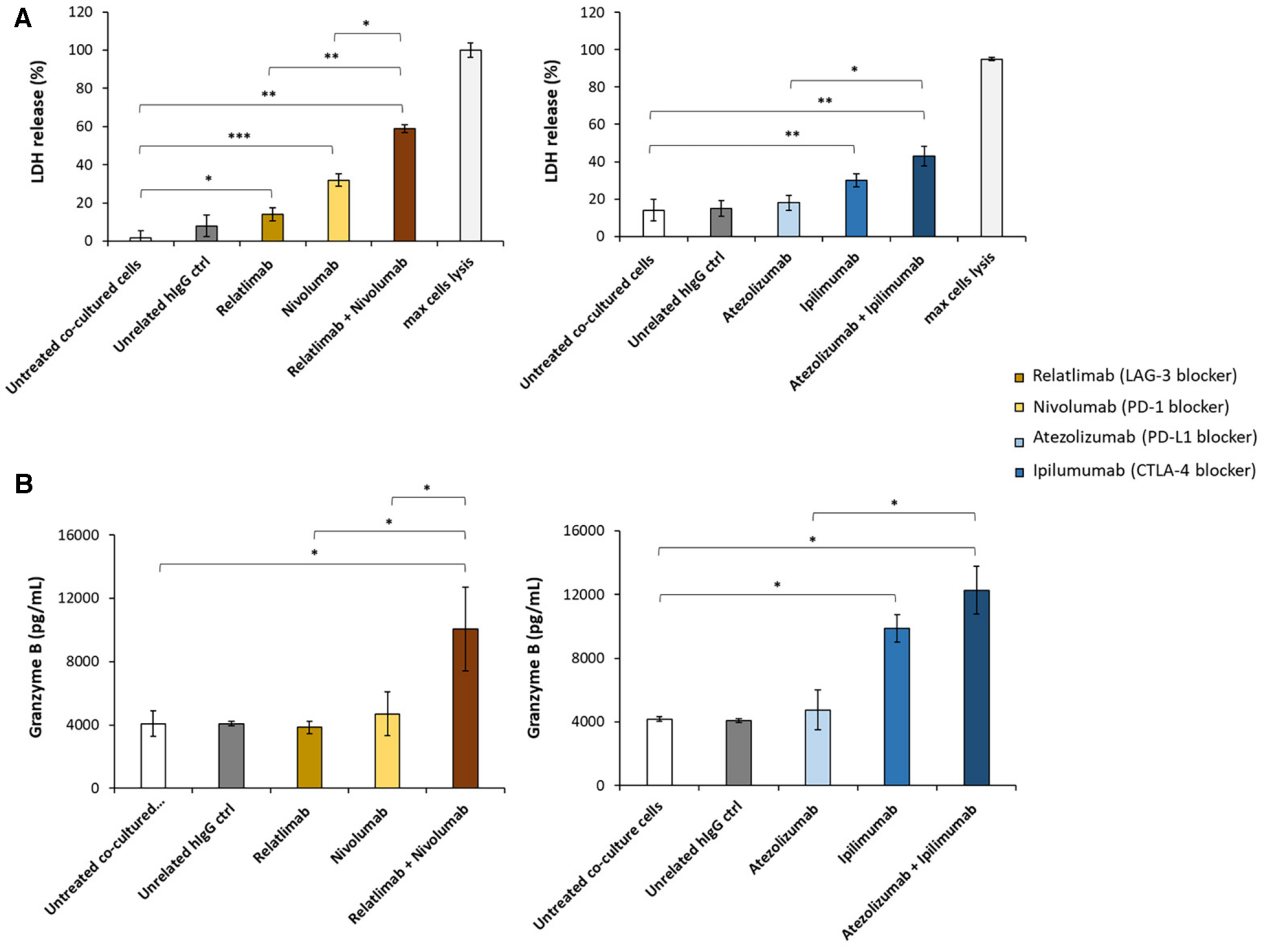
Analysis of LDH and granzyme levels released by co-cultures of HFC cells with hPBMCs. HFC cells were incubated with or without hPBMC and treated for 48 h with Relatlimab or Nivolumab (left panel) and with Atezolizumab or Ipilimumab (right panel), used at the concentration of 100 nM as single agents or in combination. (**A**) The cytotoxic effects of the mAbs on HFC cells was analyzed by measuring the levels of LDH release in the supernatants of the co-cultured cells, following the manufacturing recommendations of the CyQUANT LDH Cytotoxicity Assay by ThermoFisher. (**B**) The levels of Granzyme B were measured in the supernatants of co-cultures by using the ELISA Granzyme B kit, as described in methods. A human unrelated IgG was used as control. The values reported are related to the mean of three experiments ± SD); *p*-values were calculated by comparing each treatment with untreated co-cultured cells or the combined treatment with each single treatment and reported as ***p < 0.001; **p < 0.01; *p < 0.05.

To mimic the lymphocyte infiltration that is clinically found in cancer patients treated with ICIs who develop myocarditis, we have developed a cellular model of lymphocyte-cardiomyocyte interaction, already described in previous works (19, 22, 40) (Figure 1). To this aim, human cardiomyocyte/hPBMCs co-culture was exposed or unexposed to 100 nM Relatlimab, Nivolumab, Atezolizumab, Ipilimumab or their combinations as used in clinical trials or approved by FDA. We found that PD-L1 and CTLA-4 or PD-1 and LAG-3 blocking agents induced a higher cardiac cell lysis than the single agent treatments (see Figure 2). In fact, cells exposed to Relatlimab, Nivolumab and both in combination had a percentage of LDH release of 14 ± 3.6, 32 ± 2.1 and 59 ± 3.6%, respectively, vs. Unrelated IgG group (control; 8% ± 3.1; *p* < 0.001). Similarly, cells exposed to Atezolizumab, Ipilimumab and both in combination had a percentage of LDH release 18 ± 3.9, 30 ± 3.4 and 43 ± 5.2%, respectively, vs. Unrelated IgG group (control; 15% ± 4.0; *p* < 0.001).

**Figure 3:**
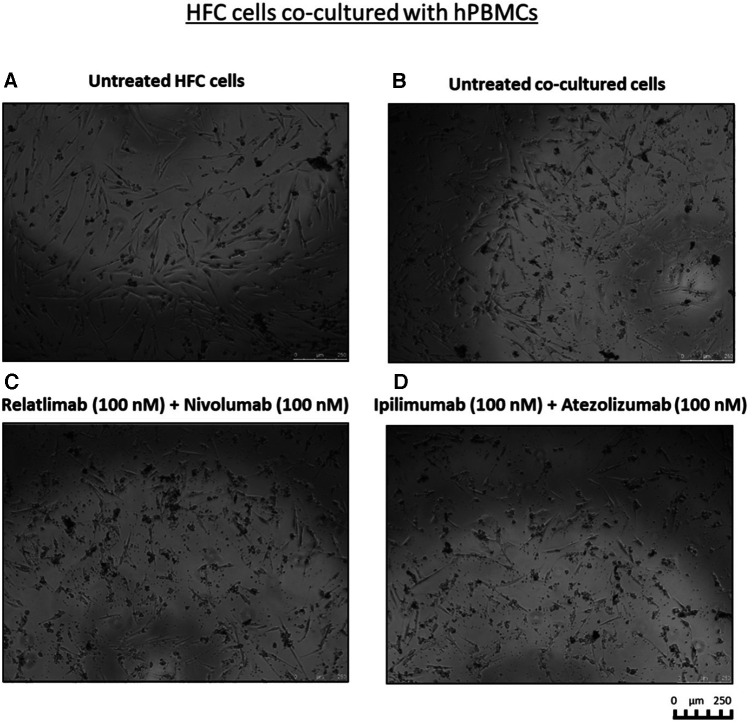
Cell debris and significant reduction in fibroblast-like phenotype of HFC cells, co-cultured with hPBMCs, exposed to combinatorial ICIs treatments. Microscopy Images of the co-cultures after treatment with combinations of Relatlimab and Nivolumab (**C**) or Ipilimumab and Atezolizumab (**D**), respectively, at the indicated concentrations. Untreated HFC cells in the absence (**A**) or in the presence of hPBMCs (**B**) were used in parallel as negative controls. The images were obtained via Leica Advanced microscopy (Leica DMI4000 B).

To clarify whether this enhanced effect was due to a higher activation of cytotoxic immune cells, we analyzed the levels granzyme B in co-culture supernatant and we found that the two combinations induced higher secretion of the cytokines, markers of T cell activation, than single agent treatments, in line with the formulated hypothesis. In details, co-cultures exposed to Relatlimab, Nivolumab and both in combination led to a granzyme B release of 3,850 ± 8, 4,705 ± 28, 10,075 ± 53% vs. Unrelated IgG group (control; 4,087 ± 16; *p* < 0.001). Similarly, cells exposed to Atezolizumab, Ipilimumab and both in combination led to a Granzyme B release of 4,750 ± 25%, 9,885 ± 34%, 12,265 ± 59% vs. Unrelated IgG group (control, 4,182,5 ± 2,66%; *p* < 0.001). Notably, Atezolizumab + Ipilimumab does not show statistically significant differences to Ipilimumab (*p* > 0.05). Moreover, as reported in Figure 3, cardiomyocytes co-cultured with hPBMC and incubated with ICIs change their cellular morphology. Some cell debris and a significant reduction in their fibroblast-like phenotype is seen, indicating cellular side effects of ICIs therapies.

### 3.2 HDAC-4 protein levels were increased in HFC cells exposed to combined immune checkpoint inhibitors

**Figure 4:**
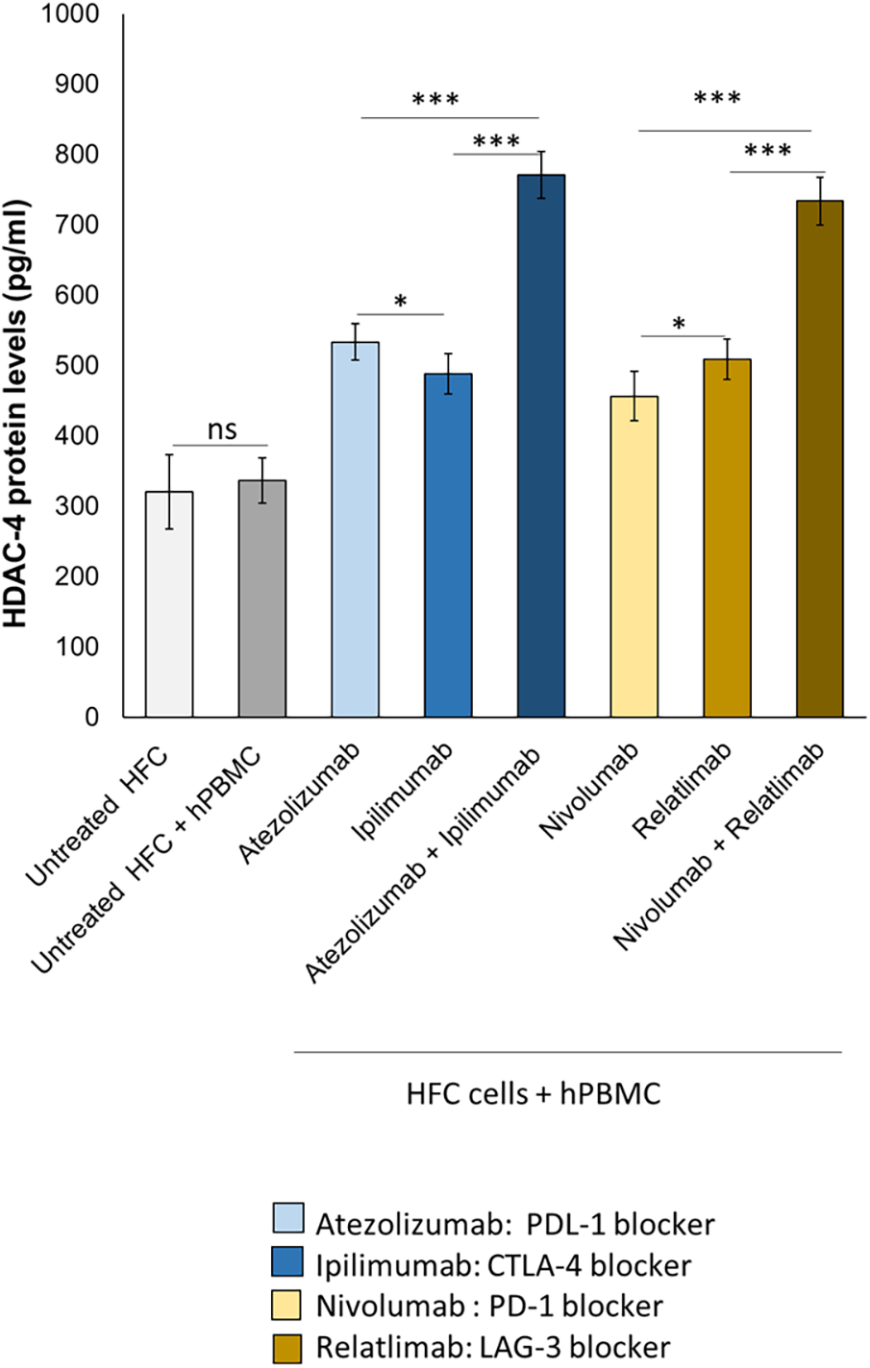
ICIs therapies increased HDAC-4 expression in HFC cells co-cultured with hPBMCs. HFC cells were incubated with or without hPBMC and treated for 48 h with Atezolizumab or Ipilimumab or Relatlimab or Nivolumab (100 nM). HDAC-4 expression (pg/ml), in HFC lysate, were quantified through selective ELISA kit, as described in methods. The data represent the mean ± SD of three independent experiments ***p < 0.001; **p < 0.01; *p < 0.05.

Histone deacetylases (HDACs) are enzymes that play a critical role in cardiac function and ischemic injury (41). Patients with heart failure and atherosclerosis have high levels of HDAC-4 expression (42). Myocardial ischemic injury resulted in increases in HDAC4 protein levels. Therefore we investigated on the effect of ICIs therapy on HDAC-4 expression in HFC cell lysates. Ipilimumab, Atezolizumab, Relatlimab and Nivolumab (in monotherapy and combinatorial therapy regimens) increased protein levels of HDAC-4 in HFC cells (Figure 4). In detail, untreated HFC cells co-cultured with hPBMC, had a HDCAC-4 expression of 336.3 ± 32.3 pg/ml. Instead, Atezolizumab, Ipilimumab and both in combination increased HDAC-4 protein levels significantly (533.6 ± 25.7; 488.3 ± 28.2; 771.1 ± 33.7 pg/ml for Atezolizumab, Ipilimumab and Atezolizumab/Ipilimumab group, respectively; *p* < 0.001 vs. control). Nivolumab, Relatlimab and both in combination increased significantly HDAC-4 expression compared to HFC/hPBMC group (456.4 ± 35.1; 508.8 ± 28.5; 733.7 ± 33.8 pg/ml for Nivolumab, Relatlimab and Nivolumab/Relatlimab group, respectively; *p* < 0.001 vs. control). This behavior is in line with other preclinical work that highlight the HDAC-4 overexpression in cardiomyocytes exposed to cardiotoxic drugs, including anthracyclines.

### 3.3 Combined immune checkpoint blockade increases intracellular Ca^++^ levels, reduces endogenous ATP production and mitochondrial functions in HFC cells

**Figure 5:**
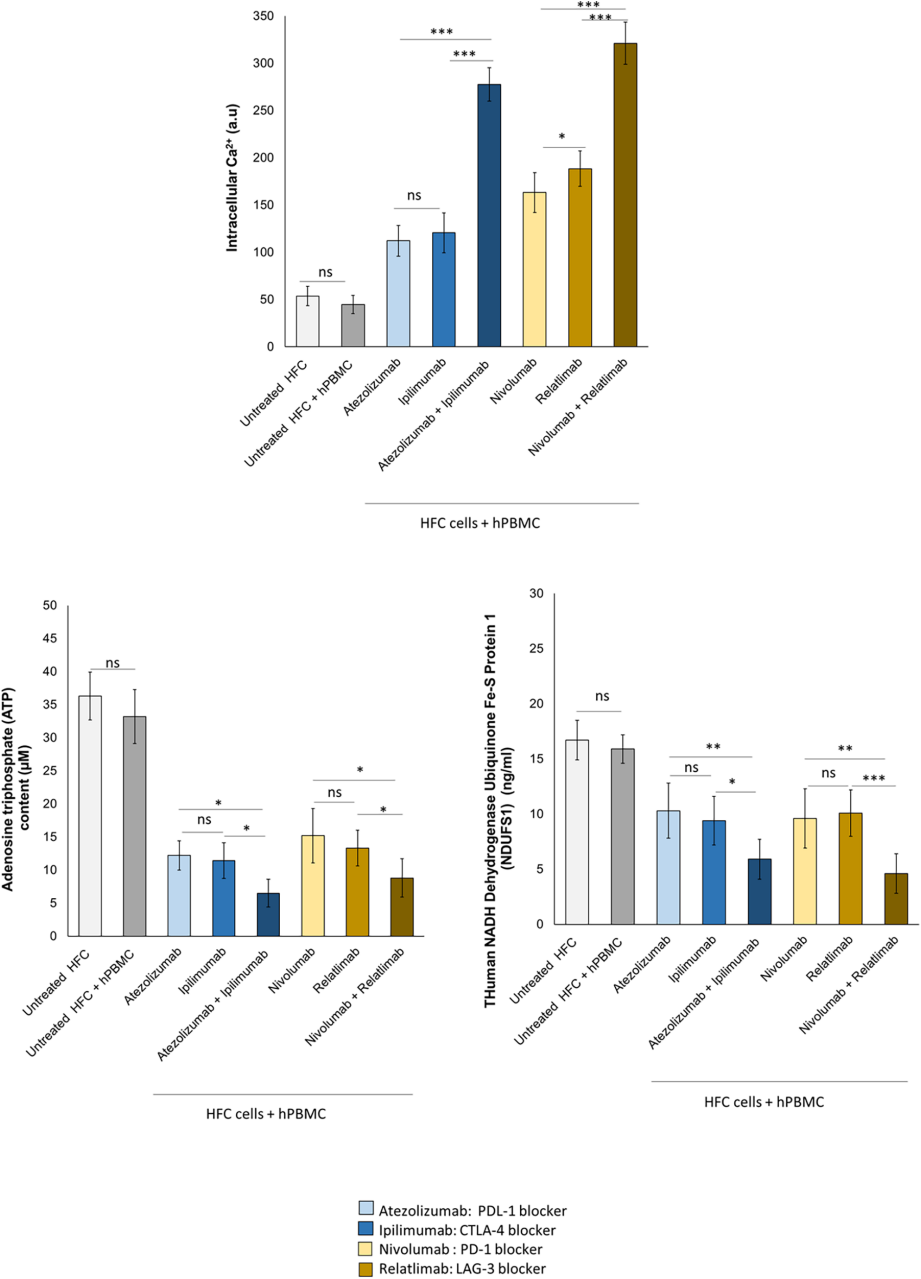
ICIs therapies increased intracellular Ca^++^ levels, decreased ATP production and NDUFS1 levels in HFC cells co-cultured with hPBMCs. HFC cells were incubated with or without hPBMC and treated for 48 h with Atezolizumab or Ipilimumab or Relatlimab or Nivolumab (100 nM). Intracellular Ca^++^ levels (a.u), in HFC lysate, were quantified through fluorescent Kit, as described in methods. Intracellular ATP (µM) and NDUFS1 (ng/ml) levels were quantified through ELISA methods, as described in methods. The data represent the mean ± SD of three independent experiments ***p < 0.001; **p < 0.01; *p < 0.05. Differences between control group (Untreated HFC + hPBMC) and ICIs groups were always statistically significant (*p* < 0.001 for all).

To study the effects of ICIs therapies on mitochondrial functions and metabolic activity of cardiomyocytes, intracellular Ca^++^ concentration, ATP content and NADH:ubiquinone oxidoreductase core subunit S1 (Ndufs1) levels were quantified. Reduced Ndufs1 levels were associated to mitochondrial dysfunctions through the reduction of ATP production in cardiomyocytes, resulting in cardiac dysfunction (36, 43). Immune checkpoint inhibitors increased intracellular calcium content in HFC lysates, indicating changes of the contractile balance of cardiomyocytes, in line with the literature data of the same cells treated with anthracyclines, trastuzumab and other cardiotoxic drugs. Combinatorial ICIs therapy significantly increases intracellular calcium concentration than monotherapy (*p* < 0.001) (Figure 5). Specifically, untreated HFC cells co-cultured with hPBMC, had an intracellular Ca^++^ content of 44.7 ± 9.6 a.u. Instead, Atezolizumab, Ipilimumab and both in combination increased intracellular Ca^++^ levels significantly (112.2 ± 16.4; 120.6 ± 21.2; 277.7 ± 17.7 a.u for Atezolizumab, Ipilimumab and Atezolizumab/Ipilimumab group, respectively; *p* < 0.001 vs. control). Nivolumab, Relatlimab and both in combination increased significantly the intracellular Ca^++^ levels compared to untreated HFC/hPBMC group (163.3 ± 21.1; 188.5 ± 18.6; 321.2 ± 22.4 a.u for Nivolumab, Relatlimab and Nivolumab/Relatlimab group, respectively; *p* < 0.001 vs. control). ATP production and Ndufs1 expression are significantly changed in HFCs exposed to ICIs, indicating mitochondrial damage induced by lymphocyte activation against cardiomyocytes under exposure to ICIs in monotherapy and combinatorial regimens (Figure 5). ATP content was drastically and significantly reduced after exposure with ICIs therapy and the same behavior was seen on Ndufs1. In detail, ATP content of untreated HFC cells co-cultured with hPBMC, was 33.2 ± 4.1 µM; instead, Atezolizumab, Ipilimumab and both in combination decreased significantly ATP content (12.2 ± 2.2, 11.4 ± 2.7, 6.5 ± 2.1 µM for Atezolizumab, Ipilimumab and Atezolizumab/Ipilimumab group, respectively; *p* < 0.001 vs. control). Nivolumab, Relatlimab and both in combination decreased significantly ATP levels compared to untreated HFC/hPBMC group (15.2 ± 4.1, 13.3 ± 2.7, 8.8 ± 2.9 µM for Nivolumab, Relatlimab and Nivolumab/Relatlimab group, respectively; *p* < 0.001 vs. control). Moreover, Ndufs1 protein levels of untreated HFC cells co-cultured with hPBMC, was 15.9 ± 1.3 ng/ml; Atezolizumab, Ipilimumab and both in combination decreased significantly Ndufs1 expression (10.3 ± 2.5, 9.4 ± 2.2, 5.9 ± 1.8 ng/ml for Atezolizumab, Ipilimumab and Atezolizumab/Ipilimumab group, respectively; *p* < 0.001 vs. control). Nivolumab, Relatlimab and both in combination decreased significantly the Ndufs1 levels compared to untreated HFC/hPBMC group (9.6 ± 2.7, 10.1 ± 2.1, 4.6 ± 1.7 ng/ml for Nivolumab, Relatlimab and Nivolumab/Relatlimab group, respectively; *p* < 0.001 vs. control).

### 3.4 Combined immune checkpoint blockade increases intracellular reactive oxygen Species and lipid peroxidation in human cardiomyocytes co-cultured with hPBMC

**Figure 6:**
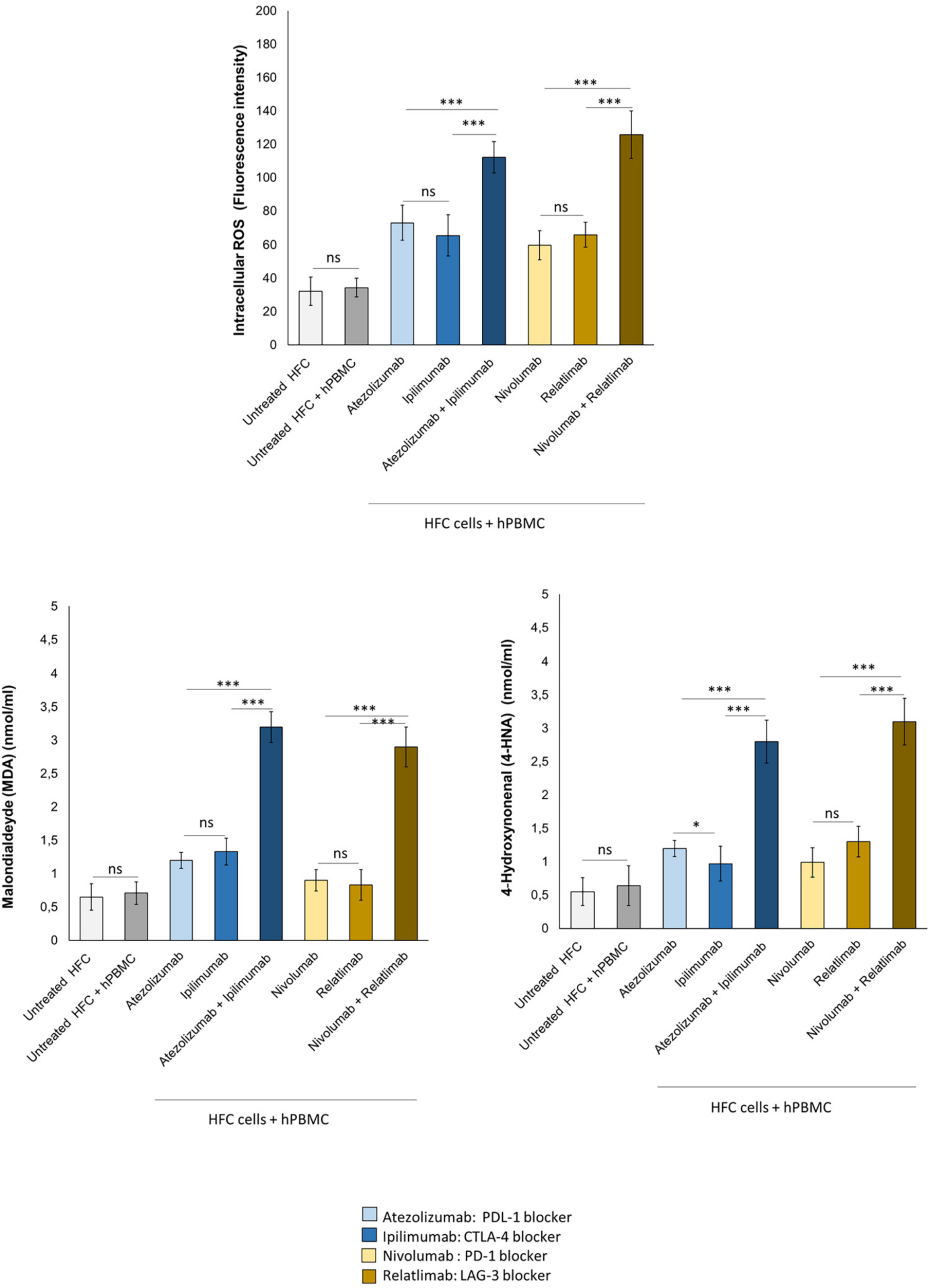
ICIs therapies increased intracellular ROS and lipid peroxidation products (MDA and 4-HNA) levels in HFC cells co-cultured with hPBMCs. HFC cells were incubated with or without hPBMC and treated for 48 h with Atezolizumab or Ipilimumab or Relatlimab or Nivolumab (100 nM). Intracellular ROS levels (fluorescence intensity), in HFC lysate, were quantified through fluorescent Kit, as described in methods. Intracellular lipid peroxidation products, such as MDA and 4-HNA (mmol/ml) levels were quantified through colorimetric assay, as described in methods. The data represent the mean ± SD of three independent experiments ***p < 0.001; **p < 0.01; *p < 0.05.

Intracellular Oxygen Species and lipid peroxidation are strictly involved in anticancer drug-mediated cardiotoxicity, including immune-mediated cardiovascular affections (19, 20). In human cardiomyocytes co-cultured with hPBMC, ICIs in monotherapy and combinatorial regimen increased significantly iROS and lipid peroxidation products MDA and 4-HNA (Figure 6). In detail, fluorescence intensity related to iROS levels in cell lysates of untreated HFC cells, co-cultured with hPBMC, was 34.4 ± 6.6 a.u; instead, Atezolizumab, Ipilimumab and both in combination increased significantly I ROS levels (73.2 ± 10.3, 65.5 ± 12.2, 112.2 ± 9.5 a.u for Atezolizumab, Ipilimumab and Atezolizumab/Ipilimumab group, respectively; *p* < 0.001 vs. control). Nivolumab, Relatlimab and both in combinatorial regimen increased fluorescence intensity related to iROS content compared to HFC/hPBMC group (59.8 ± 8.8, 66.1 ± 7.5, 125.7 ± 14.3 a.u for Nivolumab, Relatlimab and Nivolumab/Relatlimab group, respectively; *p* < 0.001 vs. control). MDA and 4-HNA levels in cell lysates of untreated HFC cells co-cultured with hPBMC, were 0.71 ± 0.17 and 0.64 ± 0.3 nmol/ml, respectively. Instead, Atezolizumab, Ipilimumab and both in combination increased drastically their levels (1.2 ± 0.12, 1.33 ± 0.2, 3.2 ± 0.23 nmol/ml for Atezolizumab, Ipilimumab and Atezolizumab/Ipilimumab group, respectively; *p* < 0.001 vs. control for MDA; 1.2 ± 0.12, 0.97 ± 0.26, 2.8 ± 0.32 nmol/ml for Atezolizumab, Ipilimumab and Atezolizumab/Ipilimumab group, respectively; *p* < 0.001 vs. control for 4-HNA). The same behavior was seen for Nivolumab, Relatlimab and both in combinatorial regimen, with a significant increase in MDA and 4-HNA levels, compared to untreated HFC/hPBMC group (0.9 ± 0.18, 0.83 ± 0.23, 2.9 ± 0.3 nmol/ml for Nivolumab, Relatlimab and Nivolumab/Relatlimab group, respectively; *p* < 0.001 vs. control for MDA; 0.99 ± 0.22, 1.3 ± 0.23, 3.1 ± 0.35 nmol/ml for Nivolumab, Relatlimab and Nivolumab/Relatlimab group, respectively; *p* < 0.001 vs. control for 4-HNA) (Figure 6).

### 3.5 Combined immune checkpoint blockade increases NLRP-3-pro-inflammatory cytokines pathways in lymphocyte-cardiomyocyte model

**Figure 7:**
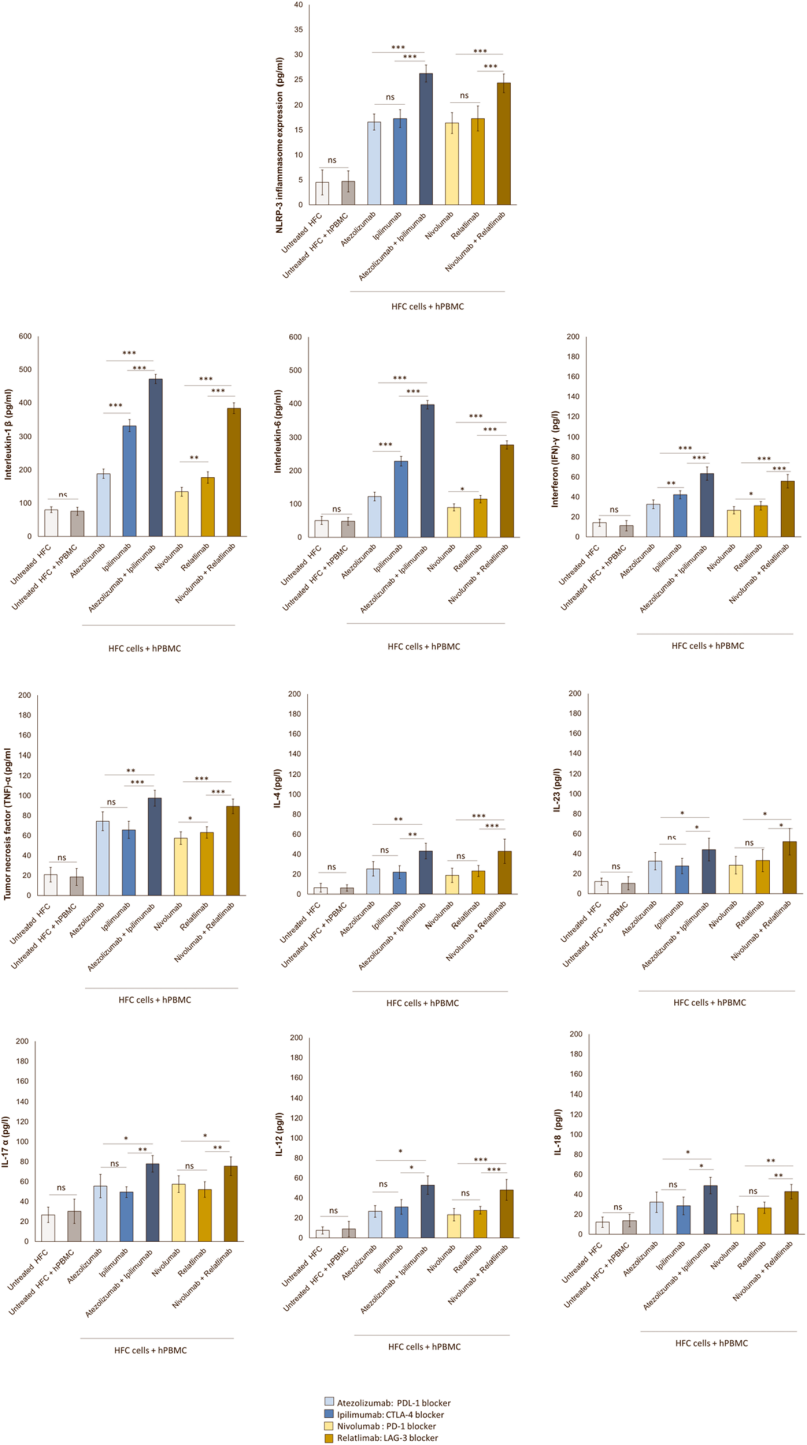
Combinatorial ICIs therapies increases NLRP3 expression and release of several pro-inflammatory biomarkers in co-cultures of HFC cells with hPBMCs. HFC cells were incubated with or without hPBMC and treated for 48 h with Atezolizumab or Ipilimumab or Relatlimab or Nivolumab (100 nM). NLRP-3 inflammasome expression (in HFC lysate), IL-1β, IL-6, TNF-α, IL-4, IL-23, IL-17a, IL-12, IL-18 and INF-γ release in surnatant (pg/ml) were quantified through selective ELISA kits, as described in methods. The data represent the mean ± SD of three independent experiments ***p < 0.001; **p < 0.01; *p < 0.05.

Therapies based on ICIs are associated to cardiac and vascular injuries that correlate with high hsCRP levels indicating systemic inflammation (44). Analyses of protein expression through ELISA method in hPBMC –cardiomyocyte models exhibited highly significant increases in NLRP-3 induced by Atezolizumab, Ipilimumab, Nivolumab and Relatlimab in monotherapy compared to untreated HFC and untreated HFC-hPBMC co-cultures (Figure 7). Interestingly, combinatorial ICIs therapies increased of five times the NLRP3 expression (*p* < 0.001 vs. monotherapy). In detail, NLRP3 protein levels in cell lysates of untreated HFC cells, co-cultured with hPBMC, were 4.7 ± 2.1 pg/ml. Instead, Atezolizumab, Ipilimumab and both in combination increased drastically NLRP3 levels (16.6 ± 1.6, 17.3 ± 1.8, 26.2 ± 1.7 pg/ml for Atezolizumab, Ipilimumab and Atezolizumab/Ipilimumab group, respectively; *p* < 0.001 vs. control). Nivolumab, Relatlimab and both in combinatorial regimen drastically increased NLRP3 protein levels than untreated HFC/hPBMC group (16.4 ± 2.1, 17.7 ± 2.5, 24.3 ± 1.8 pg/ml for Nivolumab, Relatlimab and Nivolumab/Relatlimab group, respectively; *p* < 0.001 vs. control). Considering that NLRP-3 drives cytokine storm (45), it was evaluated if ICIs therapies could enhance IL-6 and IL-1β levels. In line with NLRP-3 release results, ICIs monotherapy and especially combinatorial therapies strongly enhanced both cytokines, indicating a pro-inflammatory phenotype. In detail, IL-1β and IL-6 protein levels in supernatant of untreated HFC cells co-cultured with hPBMC, were 75.6 ± 12.2 and 47.8 ± 11.1 pg/ml, respectively. Instead, Atezolizumab, Ipilimumab and both in combination increased drastically their levels (188.5 ± 14.2, 332.4 ± 17.2, 471.7 ± 13.6 pg/ml for Atezolizumab, Ipilimumab and Atezolizumab/Ipilimumab group, respectively; *p* < 0.001 vs. control for IL-1β;122.2 ± 13.1, 228.6 ± 14.5, 396.7 ± 12.8 pg/mL for Atezolizumab, Ipilimumab and Atezolizumab/Ipilimumab group, respectively; *p* < 0.001 vs. control for IL-6). The same behavior was seen for Nivolumab, Relatlimab and both in combinatorial regimen, with a significant increase in IL-1β and IL-6 level, compared to untreated HFC/hPBMC group (134.2 ± 13.7, 177.2 ± 17.4, 383.5 ± 16.3 pg/ml for Nivolumab, Relatlimab and Nivolumab/Relatlimab group, respectively; *p* < 0.001 vs. control for IL-1β; 89.6 ± 10.6, 114.8 ± 11.5, 277.8 ± 12.3 pg/ml for Nivolumab, Relatlimab and Nivolumab/Relatlimab group, respectively; *p* < 0.001 vs. control for IL-6) (Figure 7).

Based on Tarhini et al. results (46) that ICIs increases circulating pro-inflammatory cytokines like tumor necrosis factor (TNF)-α and interferon (IFN)-γ, could induce a similar behavior in lymphocyte-cardiomyocyte models. In line with clinical data of Tarhini et al., it was found that Atezolizumab, Ipilimumab, Nivolumab and Relatlimab in monotherapy increased interferon (IFN)-γ and tumor necrosis factor (TNF)-α levels in cardiomyocytes. Evidences of synergistic pro-inflammatory effects of combinatorial ICIs regimen compared to monotherapies are seen. In details, (IFN)-γ and (TNF)-α protein levels in supernatant of untreated HFC cells co-cultured with hPBMC, was 11.2 ± 5.2 and 18.5 ± 8.55 pg/ml, respectively. Instead, Atezolizumab, Ipilimumab and both in combination increased drastically their release (32.6 ± 4.38, 42.3 ± 4.0, 63.5 ± 6.74 pg/ml for Atezolizumab, Ipilimumab and Atezolizumab/Ipilimumab group, respectively; *p* < 0.001 vs. control for IFN-γ; 74.3 ± 9.43, 65.7 ± 8.6, 97.5 ± 7.9 pg/ml for Atezolizumab, Ipilimumab and Atezolizumab/Ipilimumab group, respectively; *p* < 0.001 vs. control for TNF-α). The same behavior was seen for Nivolumab, Relatlimab and both in combinatorial regimen, with a significant increase in IFN-γ and TNF-α release than untreated HFC/hPBMC group (26.7 ± 3.67, 31.2 ± 4.22, 55.8 ± 6.8 pg/ml for Nivolumab, Relatlimab and Nivolumab/Relatlimab group, respectively; *p* < 0.001 vs. control, relatively to IFN-γ; 57.5 ± 6.3,63.2 ± 5.77, 89.4 ± 7.32 pg/ml for Nivolumab, Relatlimab and Nivolumab/Relatlimab group, respectively; *p* < 0.001 vs. control, relatively to TNF-α) (Figure 7). The same behavior was seen for other pro-inflammatory cytokines involved in ICIs-mediated myocardial affections, such as IL-4, IL-23, IL-17a, IL-12 and IL-18 that, in line with literature (33), are significantly enhanced after ICIs therapy due to the induced immune-mediated pro-inflammatory phenotype.

### 3.6 Combined immune checkpoint blockade increases H-FABP, troponin-T, BNP and NT-pro-BNP levels in cardiomyocyte-lymphocyte models

**Figure 8:**
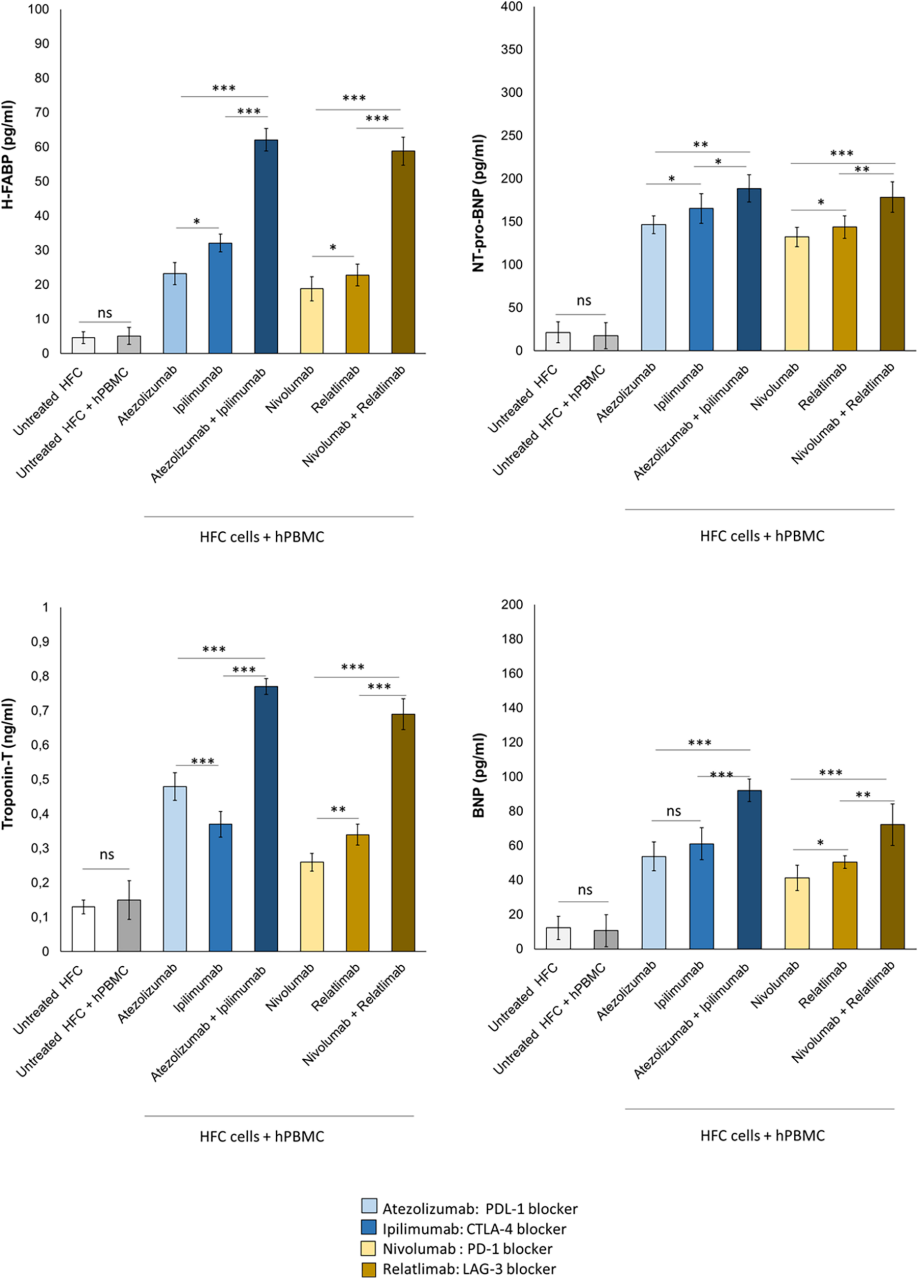
Combinatorial ICIs therapies increase cardiotoxicity biomarkers in co-cultures of HFC cells with hPBMCs. HFC cells were incubated in the absence or in the presence of human lymphocytes and treated for 48 h with Atezolizumab or Ipilimumab or Relatlimab or Nivolumab at 100 nM as single agents or in combination. H-FABP expression (pg/ml), NT-pro-BNP (pg/ml), Troponin T (ng/ml)and BNP (pg/ml) expression were quantified in co-cultures through selective ELISA kits, as described in methods. The data represent the mean ± SD of three independent experiments ***p < 0.001; **p < 0.01; *p < 0.05.

Patients with high levels of H-FABP are exposed to increased risk of death and cardiomyopathies; Troponin T, BNP and NT-pro-BNP are established biomarkers of cardiotoxicity induced by anthracyclines, immune checkpoint inhibitors and radiotherapy (47, 48). Therefore, it was studied if combined immune checkpoint blockade could increase the secretion of H-FABP, Troponin-T, BNP and NT-pro-BNP form cardiac cells (Figure 8). First, Atezolizumab, Ipilimumab, Nivolumab and Relatlimab in monotherapy were able to increase significantly all biomarkers of cardiotoxicity; specifically, H-FABP levels were 23.2 ± 3.2, 32.1 ± 2.6 and 62.1 ± 3.3 pg/ml for Atezolizumab, Ipilimumab and Atezolizumab/Ipilimumab groups, respectively; *p* < 0.001 vs. control; 18.8 ± 3.5, 22.8 ± 3.2, 58.8 ± 4.1 pg/ml for Nivolumab, Relatlimab and Nivolumab/Relatlimab groups, respectively; *p* < 0.001 vs. control. Moreover, NT-pro-BNP levels were 146.5 ± 10.4, 165.4 ± 17.3 and 188.6 ± 15.9 pg/ml for Atezolizumab, Ipilimumab and Atezolizumab/Ipilimumab groups, respectively; *p* < 0.001 vs. control; 132.2 ± 11.2, 143.7 ± 13.2 and 178.5 ± 17.8 pg/ml for Nivolumab, Relatlimab and Nivolumab/Relatlimab groups, respectively; *p* < 0.001 vs. control.Therefore, also in this case, Atezolizumab associated to Ipilimumab and Nivolumab associated to Relatlimab exerted synergic cardiotoxic properties with a significant enhancement of NT-proBNP and H-FABP compared to monotherapies (Figure 8).

The same behavior was seen for Troponin-T and BNP: in brief, Atezolizumab, Ipilimumab, Nivolumab and Relatlimab in monotherapy were able to increase significantly all biomarkers of cardiotoxicity; specifically, Troponin-T levels were 0,48 ± 0.04, 0.37 ± 0.037 and 0.77 ± 0.023 ng/ml for Atezolizumab, Ipilimumab and Atezolizumab/Ipilimumab groups, respectively; *p* < 0.001 vs. control; 0,26 ± 0.025, 0.34 ± 0.04 and 0.69 ± 0.045 ng/ml for Nivolumab, Relatlimab and Nivolumab/Relatlimab groups, respectively; *p* < 0.001 vs. control. Moreover, BNP levels were 53.7 ± 8.4, 61.1 ± 9.3 and 92.1 ± 6.6 pg/ml for Atezolizumab, Ipilimumab and Atezolizumab/Ipilimumab groups, respectively; *p* < 0.001 vs. control; 41.2 ± 7.3, 50.4 ± 3.7 and 72.2 ± 12.1 pg/ml for Nivolumab, Relatlimab and Nivolumab/Relatlimab groups, respectively; *p* < 0.001 vs. control (Figure 8).

## 4 Discussion

Cancer cells exerts immune evasion through several mechanisms, including the overexpression of cytotoxic T-lymphocyte antigen 4 (CTLA-4), Programmed death 1 (PD-1), or Lymphocyte-activation gene 3 (LAG-3) (49, 50). Immunotherapy involves the use of ICIs that increases the immune recognition of tumor cells through the selective inhibition of peripheral immune tolerance in tumor tissue (51). ICIs involves mainly CTLA-4, PD-1, PD-L1 or LAG-3 blocking agents that reactivates the immune system against tumour cells (52). In brief, the reduction of peripheral immune tolerance allows the immune-related reactivity against cancer and non cancer cells, including myocardial, thyroid and intestine cells (52). The proper expression of PD-1/PDL-1 pathway in peripheral tissues, including the heart, pituitary gland, liver, thyroid and intestinal mucosa determines lymphocyte anergy and limits their potential self-reactivity (53). In fact, cancer patients treated with ICIs-based therapies frequently report cases of auto-immune reactions, including mucositis, thyroiditis, pituitarism, osteoarthritis and, more rarely, myocarditis (53). Given that the PD-1/PDL-1 pathway is crucial in cardiac immune tolerance, the use of immunotherapies results in the accumulation of autoreactive CD-3^+^/CD-8^+^ lymphocytes in myocardial tissue, exposing patients to high risk of inflammatory myocarditis (54).

Intratumor lymphocytes express high quantities of PD-1 but also of LAG-3, another surface molecule with inhibitory activity on the immune system (55, 56). PD-1 and LAG-3 co-localize on the lymphocyte membrane; moreover, LAG-3 also co-localizes with galectin-3 causing both inhibition of CD-4 lymphocytes and activation of regulatory T lymphocytes, with consequent peripheral immune tolerance (57). Preclinical studies have shown that PD-1 and LAG-3 blocking agents have synergistic antitumor activity compared to monotherapies (58). The first anti LAG-3 antibody for clinical use is Relatlimab which is able to reactivate T lymphocytes in tumor tissues in a significant way (59). Two main randomized clinical trials have investigated the effects of Relatlimab in combination with Nivolumab in cancer patients: in a study on patients with melanoma refractory to therapy with PD-1 blocking agents, the combinatorial use of Relatlimab with Nivolumab showed significant antitumor efficacy with increased survival (60). A double blind randomized clinical trial, called Relativity-047, combined Relatlimab with Nivolumab in metastatic or unresectable melanoma patients (61). The use of combinatorial ICis therapy based on anti LAG-3 and anti PD-1 was approved for clinical use in the United States in March 2022 and in Europe a few months later (62). Another recently approved combinatorial therapy for the treatment of metastatic or advanced Non Small Cell Lung Cancer is the association of anti PDL-1 with anti CTLA-4. This recent combination also showed significant increases in survival compared to monotherapy (63).

The new ICIs-based combinatorial therapies increase the incidence of immune-related adverse events compared to monotherapy regimens (64). The most common ICIs-related events are dyspnea, cough, pneumonitis, hypothyroidism, hyperthyroidism, uveitis, hemolytic anemia, neutropenia, myositis, neuropathy and vasculitis (64). Moreover, as recently summarized in JACC, ICIs-related cardiotoxicity events are seen in cancer patients, involving myocarditis, pericarditis, arrhythmias, heart failure and conduction diseases (64). In a very recent network meta-analysis of cardiovascular events in ICIs-treated lung cancer patients authors highlight on the higher incidence of myocarditis, pericarditis and vasculitis in CTLA-4 blocking agents associated to chemotherapy or PDL-1 therapy regimen compared to monotherapy, indicating mechanisms of cardiotoxic synergism in combinatorial ICIs therapy (65). In another study, PD-1 and CTLA-4 blocking agents increased adverse cardiac events compared to monotherapy regimen. Another study associates Nivolumab and Ipilimumab observing an over four-fold increased risk of vasculitis and myocarditis compared to ICI monotherapy (66).

ICIs-related cardiovascular events are due to several mechanisms, involving the breakdown of peripheral immune tolerance (67). More in detail, peripheral immune tolerance is partially mediated by the expression of CTLA-4 that competes with CD-28 to inhibit T-lymphocyte activation and proliferation, preventing immune response in heart tissue through MHC type I-TCR pathways. Moreover, PD-1/PDL-1 pathway is a key driver of immune tolerance against heart tissue through the induction of anergic T-lymphocytes (68). Administration of CTLA-4 or PD-1 or LAG-3 blocking agents activates T-lymphocytes in myocardial tissues that inhibit the peripheral immune tolerance.

**Figure 9:**
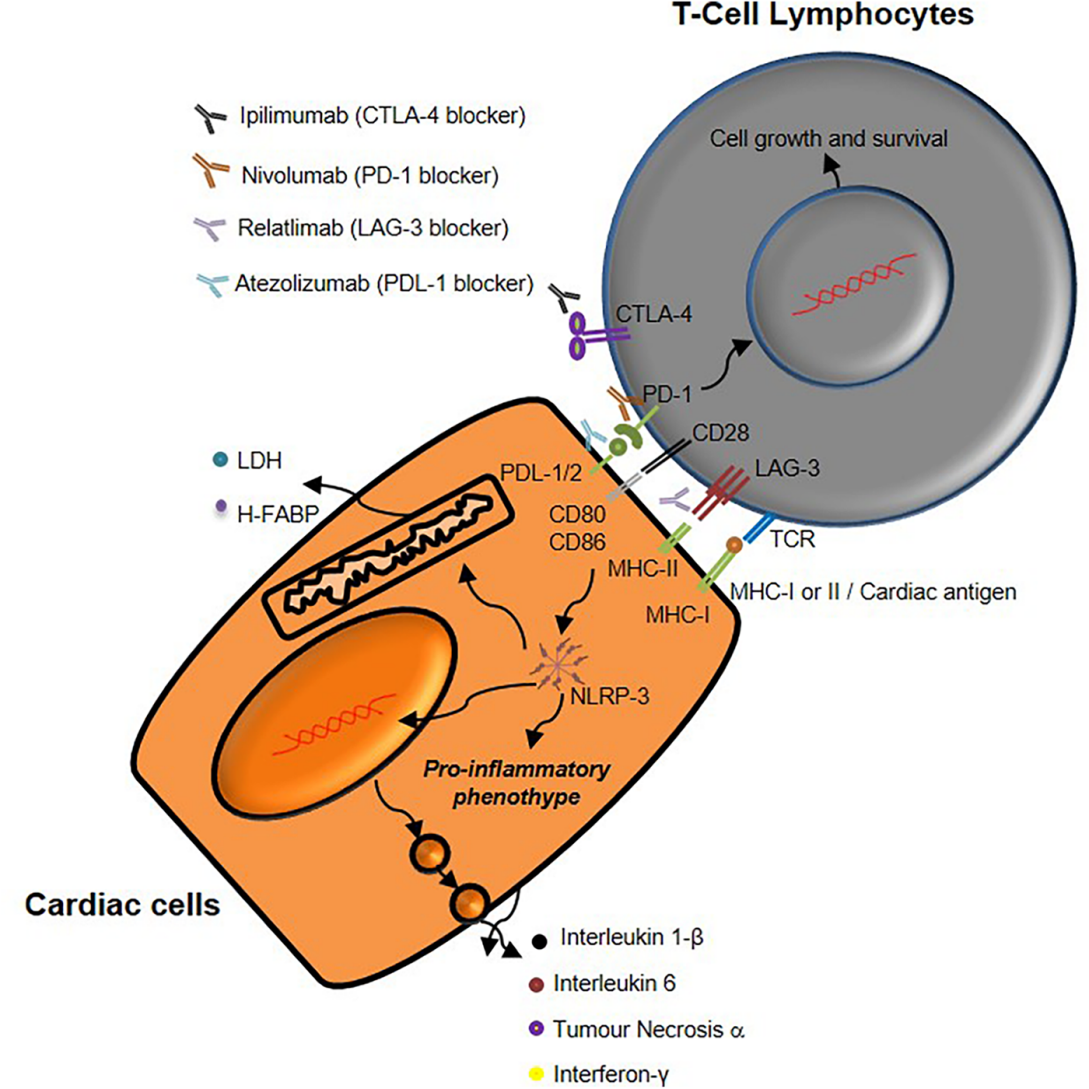
Schematic representation of mechanisms underlying immune-related cardiac toxicity due to CTLA-4, PD-1 and LAG-3 blocking agents therapy.

The current study aimed to evaluate the pro-inflammatory effects of short-term ICIs treatment in co-cultures of hPBMC and human cardiomyocytes (Figure 9). As stated before, the current clinical data indicates that only a small percentage of patients experienced cardiovascular events after combinatorial ICIs therapy or ICIs + chemotherapy/radiotherapy regimen but no preclinical and clinical evidences are, to the best of our knowledge, reported in literature on the anti LAG-3 antibody in monotherapy or combinatorial therapy. Notably, PDL-1 is expressed in cardiac cells as well as in the same cell line used in this study (40).

Data of this study indicate that combinatorial immune checkpoint blockade, especially including anti-LAG-3 monoclonal antibody, induces a pro- inflammatory phenotype in lymphocyte-cardiac cells models, thus indicating that these therapies should be closely monitored by the multidisciplinary team consisting of oncologists, cardiologists and immunologists (Figures 3, 4). LAG-3 is mainly expressed in immune cells with inhibitory processes of immune reaction (induction of peripheral tolerance) therefore the incubation with mAb anti LAG3 increases the reaction of lymphocytes against cardiac cells thereby in clinical scenario some immune reaction in myocardial tissue could be seen. A point of attention should be made also in anti-LAG-3 treated cancer patients.

Notably, results indicates that NLRP-3 mediated pathways are involved in combinatorial ICIs –mediated cardiotoxic events; these data are in line with literature describing NLRP-3 as key driver of myocarditis, heart failure and arrhythmias (19, 20). LDH release from cardiac cells was significantly increased in PDL-1/CTLA-4 and PD-1/LAG-3 blocking agents, compared to monotherapies. Biomarkers of cardiotoxicity, including Troponin-T, BNP, NT-Pro-BNP and H-FABP, were also strongly increased in combinatorial ICIs groups compared to monotherapies, indicating additive cardiotoxicity.

In a recent position statement from the Cardio-Oncology Study Group of the Heart Failure Association and the Cardio-Oncology Council of the European Society of Cardiology (21), the authors suggested a proper surveillance of ICIs-mediated cardiotoxicity in patients at low, medium and high cardiovascular risk. The position statement recommends that before ICI therapy, all patients should follow a cardiological evaluation with ECG and echocardiography with quantification of troponin, BNP or NT-proBNP. For patients at high cardiovascular risk, cardiological evaluation associated to the dosage of troponins, BNP or NT-proBNP are recommended, before, after two, three and four doses of ICI-therapy (21). Notably, some trials has recently shown changes in some cytokines, like IL-6 in the blood of patients treated with ICIs, correlating theme with anticancer response and cardiovascular effects in patients with non small cell lung cancer (69–71). Other recent research focalized on the role of systemic biomarkers of ICIs-mediated cardiovascular events, including hsCRP (72) although the authors conclude that large observational studies on short-, medium- and long-term toxicities should be done.

Despite the limitations of an *in vitro* study, the lymphocyte-cardiomyocyte interaction model, that has been set up also by other groups and previously used for testing other combinations of mAbs (19, 20, 22, 73), showed experimental evidences, for the first time, of potential cardiotoxicity and inflammation not only related to PDL-1 or PD1 or CTLA-4 blocking agents but also related to Relatlimab in monotherapy and combinatorial regimen with Nivolumab also. It is crucial to specify that the present model indicates that the interaction of lymphocytes and cardiomyocytes determine a pro-inflammatory phenotype through the induction of pro-inflammatory cytokines produced by cardiac cells. In fact, monocultures of HFC or hPBMC alone exposed to ICIs in monotherapy or combinatorial regimen (Supplementary Figure 1) did not change significantly the concentration of pro-inflammatory cytokines in supernatant, indicating the key role of the immune interaction between the lymphocyte and the cardiomyocyte. To the best of our knowledge, these are the first evidences of LAG-3 blocking agent-related toxicity against cardiomyocytes. These results are to be taken with extreme caution due to the limitation of an “*in vitro*” model, but place a cardiological focus on preclinical and clinical studies to be carried out in patients enrolled in immunotherapy regimen with anti LAG-3 antibodies in order to prevent adverse cardiac events induced by inflammatory-related damages. Notably, further gene expression studies are required to know the epigenetic effects of ICIs therapies in myocardial tissues through PCR and derived PCR methods. On the basis of these data, in line with recent position statement of American Heart Association, we suggest to quantify significant blood markers and time points useful for monitoring cardiotoxicity in clinical trials.

## Data Availability

The datasets presented in this study can be found in online repositories. The names of the repository/repositories and accession number(s) can be found below: https://zenodo.org/record/7940199.
