# Supplementary material for: Combinatorial immune checkpoint blockade increases myocardial expression of NLRP-3 and secretion of H-FABP, NT-Pro-BNP, interleukin-1β and interleukin-6: biochemical implications in cardio-immuno-oncology
Source: Front Cardiovasc Med. 2024 Jan 23;11:1232269. doi: 10.3389/fcvm.2024.1232269 (PMC10844473; doi:10.3389/fcvm.2024.1232269)
Supplement: Supplementary file 1 [file Table1.docx]

Supplementary Fig.1


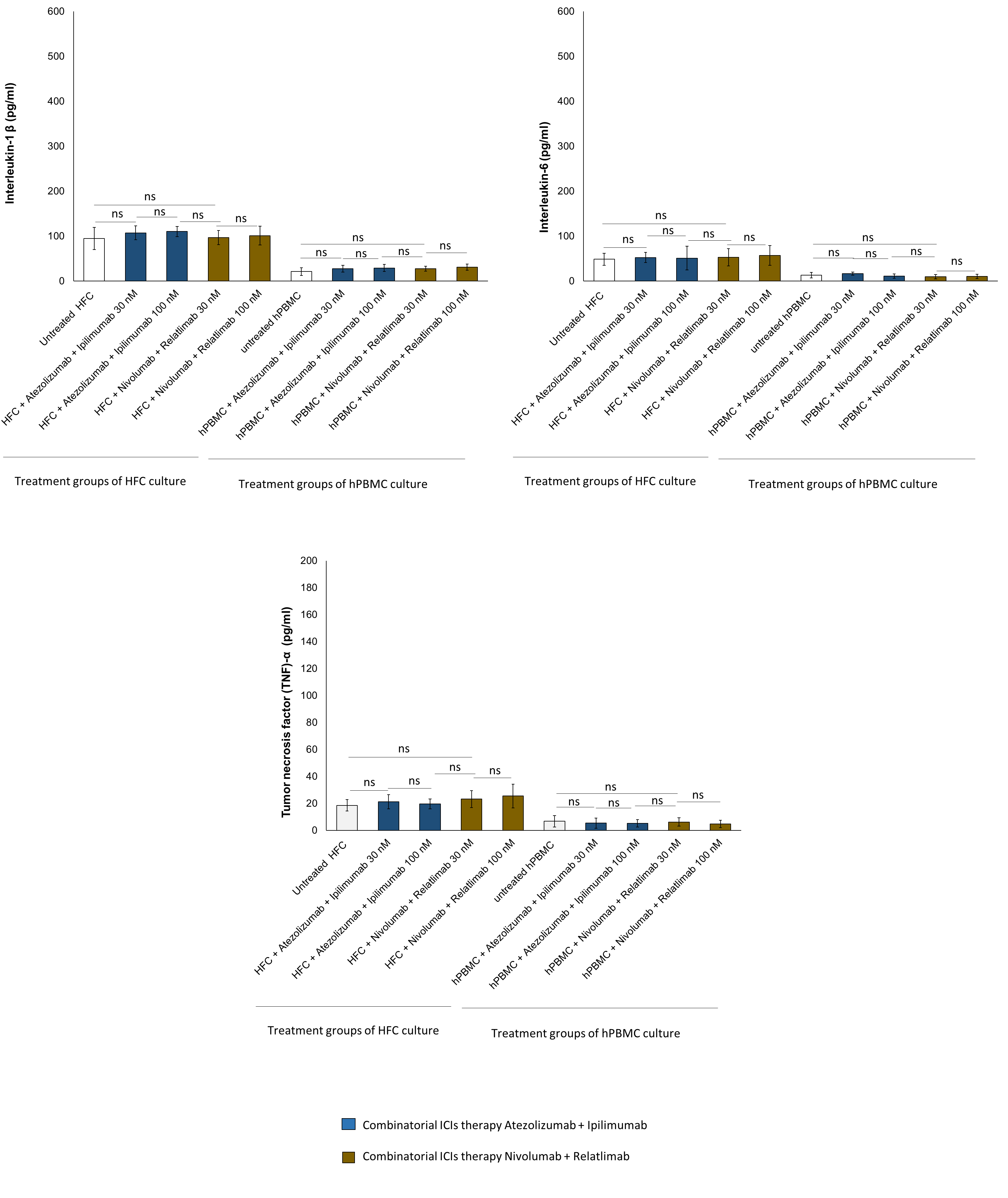


Suppl.Fig1: **Pro-inflammatory cytokine release is not affected by combinatorial ICIs therapy in cell monocultures of HFC cells or hPBMCs.** HFC cells or hPBMC were exposed for 48 hours with Atezolizumab/ Ipilimumab or Relatlimab/Nivolumab (30 and 100 nM). Pro-inflammatory cytokines, such as Interleukin 1, Interleukin 6, and TNF-a (pg/ml) were quantified in the supernatants of co-cultures by using selective ELISA kits, as described in methods. The data represent the mean ± SD of three independent experiments *** p < 0.001; ** p < 0.01; * p < 0.05.
